# A rare presentation of hepatic volvulus with resulting gastric outlet obstruction and transverse colon volvulus: a case report

**DOI:** 10.1093/jscr/rjag1035

**Published:** 2026-11-10

**Authors:** Sarah N Bunker, Yamuna T Carey, Matthew F Mikulski

**Affiliations:** Department of Surgery, University of Massachusetts Chan Medical School – Baystate Medical Center, 759 Chestnut Street, Springfield, MA 01199, United States; Division of Trauma, Acute Care Surgery and Surgical Critical Care, University of Massachusetts Chan Medical School – Baystate Medical Center, 759 Chestnut Street, Springfield, MA 01199, United States; Department of Surgery, University of Massachusetts Chan Medical School – Baystate Medical Center, 759 Chestnut Street, Springfield, MA 01199, United States

**Keywords:** hepatic volvulus, hepatopexy, nonrotation, transverse colon volvulus, case report

## Abstract

Surgeons often hear about bowel volvulus, though hepatic volvulus is less often seen in practice. Hepatic volvulus has been seldom defined, though may be related to hepatoptosis (“wandering liver”) in which there is excessive hepatic mobility due to abnormalities of the standard anatomic hepatic fixation. Torsion of the liver can result in ischemic injury to the liver or secondary obstruction of the colon. Patients that have been described to have hepatic volvulus may present with abdominal pain and associated bowel obstruction, primarily of the transverse colon. Given our patient’s worsening physical exam, he was taken to the operating room for an exploratory laparotomy. He underwent hepatopexy and extended right hemicolectomy with ileocolic anastomosis to prevent the risk of re-volvulus. A gastrostomy tube was placed for gastropexy. This operative approach has not been well studied, nor performed, making this a novel approach to a rare presentation.

## Introduction

Hepatic torsion from abnormal hepatic ligaments is a rarely seen pathology seldom described in the literature [1, 2]. Hepatic volvulus operative reports from 1970 to 2023 note findings of colonic volvulus as well [1, 2]. Patients may present with bowel obstruction secondary to liver torsion around its vascular pedicle, leading to transverse colon volvulus and possible ischemia. Given risk of re-volvulus, operative intervention has been suggested, though rarely described [10]. Transverse colon volvulus is rare, affecting 0.93% of patients with colonic volvulus [3]. Between 1932 and 2021 only 317 cases described [4]. Transverse colon volvulus may be related to prior abdominal surgeries, congenital malformations, and chronic constipation [1]. Our patient had an omphalocele repair, developmental delay and was nonverbal. With his surgical history and prior colonic and hepatic volvulus, he was at high risk for re-volvulizing. The recommended treatment for transverse colon volvulus is surgery given high risk of recurrence and death (up to 35%) [1, 5]. Few cases reported described hepatopexy done by suturing the round ligament to the right abdominal wall, with concurrent colon resection though there is no standard approach [5].

## Case report

Our patient is a 56-year old nonverbal male with a history of diastolic heart failure, type 2 diabetes mellitus, cerebral palsy with developmental delay, and a remote history of omphalocele with surgical repair in childhood. He had recurrent admissions for small bowel obstructions and on 11/8/2024 had a computed tomography (CT) abdomen and pelvis with IV contrast demonstrating medial displacement of the liver from right colon volvulus. A repeat CT scan on 11/11/24 showed resolution of this liver medialization after colonic decompression. He was discharged without surgical intervention. He then presented on 5/28/26 with abdominal pain reported by group home staff. A CT abdomen and pelvis with IV contrast was notable for volvulus of the liver into the left hemiabdomen and transverse colon and gastric dilation secondary to hepatic volvulus. A nasogastric tube was placed. His abdominal exam was benign; thus he was admitted for observation.

On morning rounds 4 h later, he had worsening tenderness. He was hemodynamically normal and labs within normal limits though given change in physical exam, we proceeded to the operating room 9 h after admission. Intraoperatively his stomach was noted to be massively dilated presumed to be secondary to chronic obstruction from intermittent hepatoptosis (Fig. 1). The liver was exceedingly mobile without ligaments anchoring it to the diaphragm or abdominal wall (Fig. 2). The was apparent transverse colon dilatation and redundancy, secondary to chronic, intermittent colon volvulus. The descending colon was relatively normal though with lax attachments to the retroperitoneum, the sigmoid colon was redundant.

**Figure 1:**
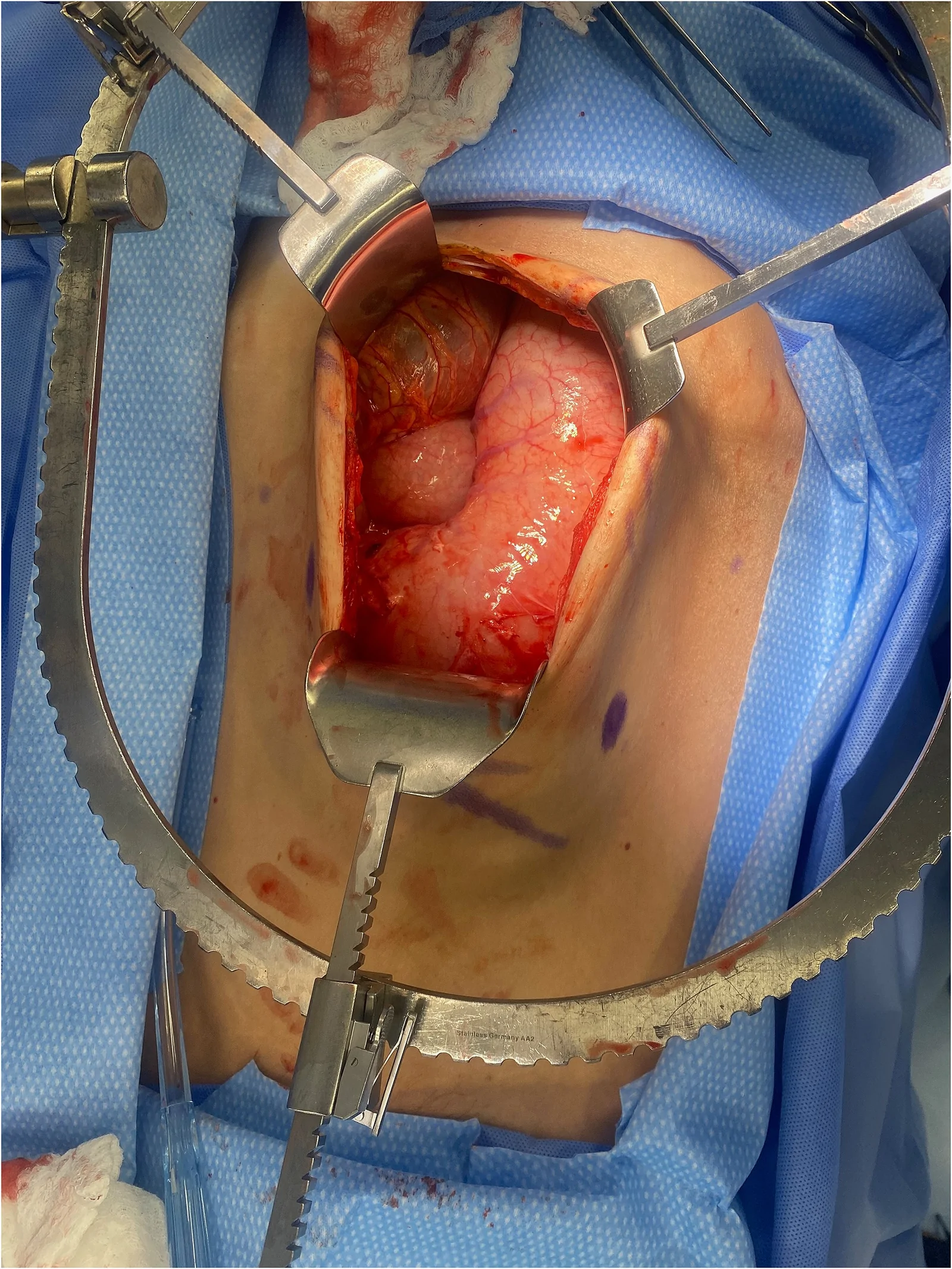
Upon entering the abdomen, we noted severe gastric dilation.

**Figure 2:**
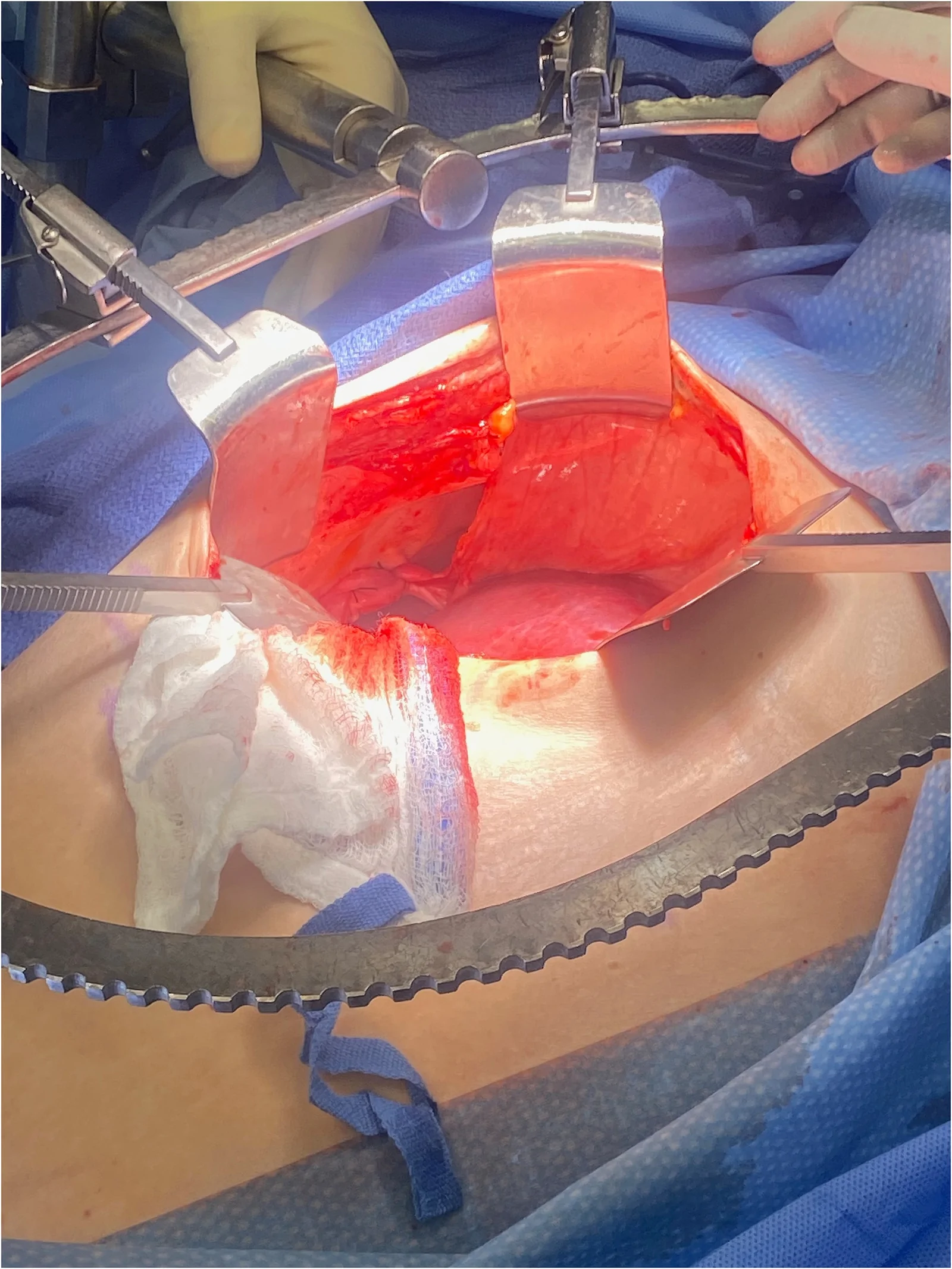
Floppy falciform and ligamentum teres noted anterior to the liver without additional ligaments securing the liver in place.

We performed a hepatopexy by suturing the ligament teres to the right lateral abdominal wall with 2-0 silk horizontal mattress sutures. A total of 5 cm of the falciform ligament was removed to correct its laxity and fixed to the anterior abdominal wall with 2-0 silk sutures in Cannell fashion, ensuring that fascia was included in the stitch (Figs 3 and 4). Decision was made to perform an extended right hemicolectomy. The splenic flexure was mobilized and a side-to-side stapled ileocolic anastomosis using 80 3.8 mm gastrointestinal anastomosis (GIA)stapler was completed. An open gastrostomy tube was placed to pexy the stomach using Stamm fixation with 3-0 silk sutures. Postoperatively he remained nothing by mouth as we awaited return of bowel function. On postoperative day three he was distended; G tube was vented for decompression. He had return of bowel function on postoperative day four and increasing tenderness on day five. A CT abdomen and pelvis with IV and PO contrast to evaluate for an underlying intra-abdominal process. The liver was in appropriate anatomic position with residual small bowel and colonic dilation. There was evidence of pneumoperitoneum, secondary to his postoperative state, no contrast extravasation was noted. Decision was made to monitor his exam.

**Figure 3:**
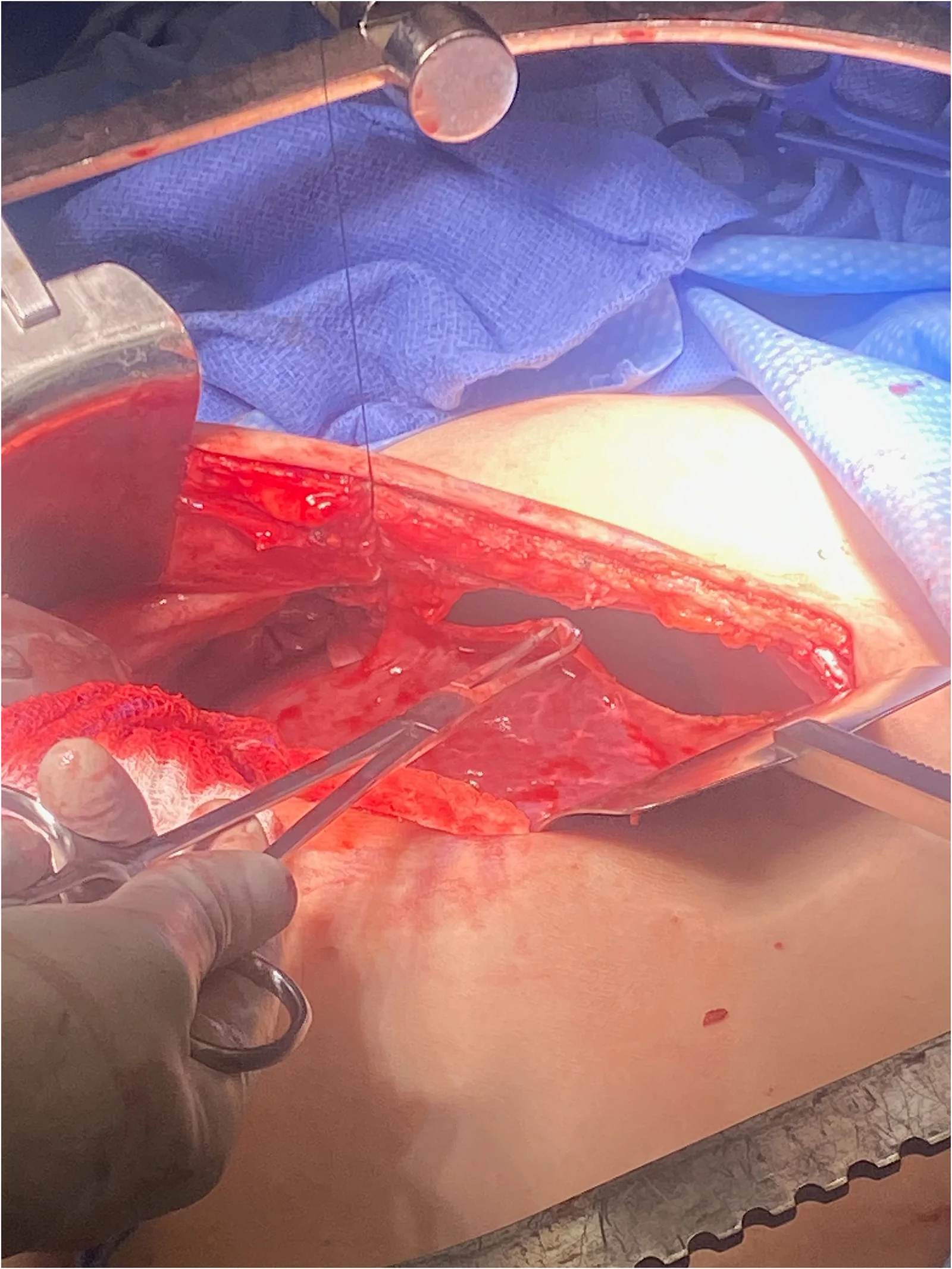
Hepatopexy being performed, the falciform ligament is being sutured to the anterior abdominal wall.

**Figure 4:**
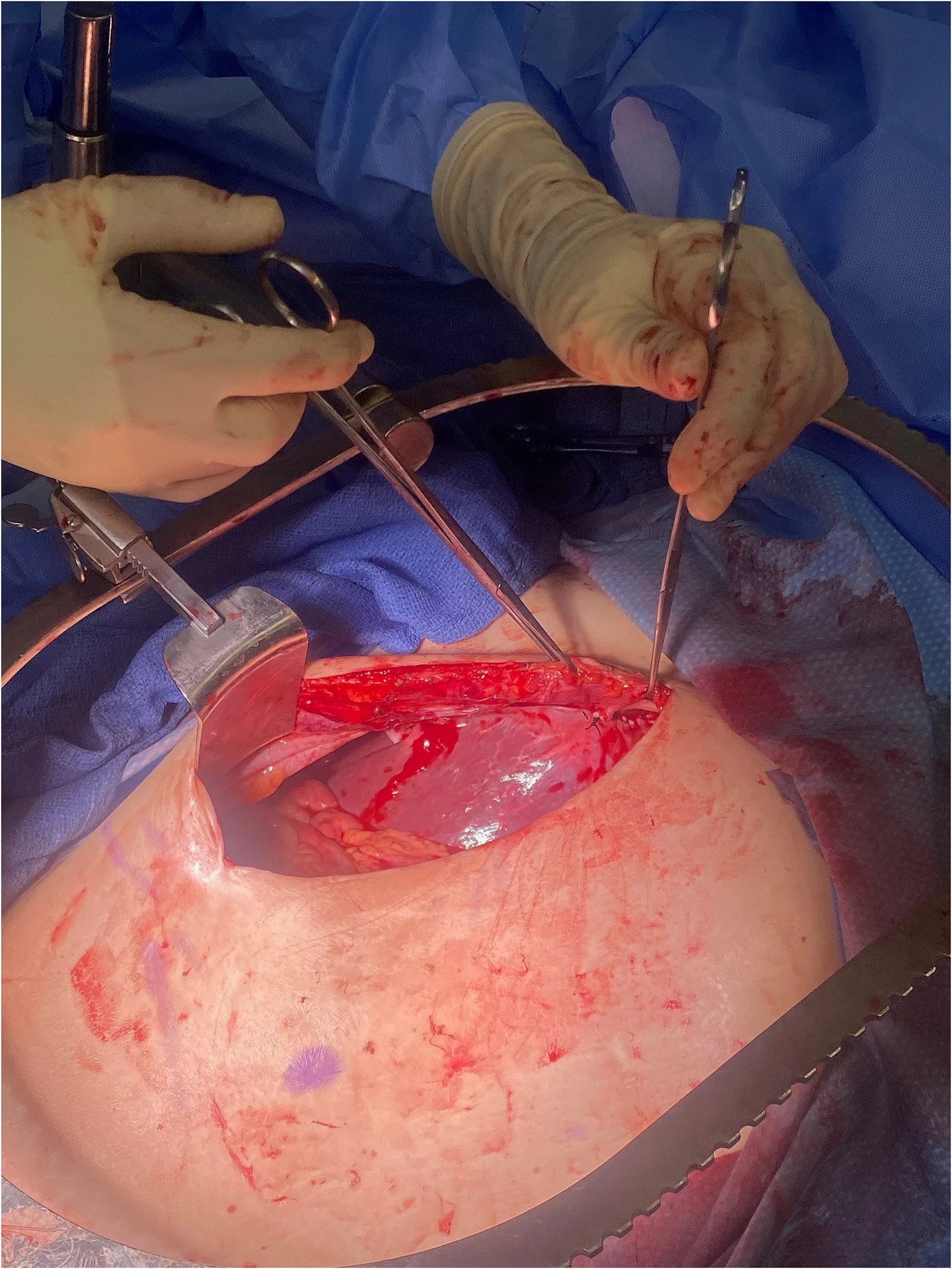
Final hepatopexy with the liver secured to the anterior abdominal wall.

On postoperative day six he was acutely hypotensive, tachycardic and somnolent. Labs demonstrated leukopenia of 0.2 and elevated lactate of 4.3. He was persistently hypotensive despite intravenous fluids with systolic blood pressures between 72 and 89, and subsequently dropped to systolics 51 and 58. Norepinephrine was started and he was upgraded to the Intensive Care Unit. A kidneys, ureter, bladder (KUB) film and upright chest XR were obtained, demonstrating gastric distention below the left hemidiaphragm and a displaced and dilated colon under the right hemidiaphragm. His health care proxy was contacted, who elected to make him comfort measures only with no escalation in care. He passed away on postoperative day seven. The family declined an autopsy.

## Discussion

Hepatic volvulus is a unique condition that may never be encountered in a surgeon’s career. We performed a hepatopexy by securing the falciform ligament to the anterior abdominal wall and the ligamentum teres to the right lateral abdominal wall, a right hemicolectomy with ileocolic anastomosis, and gastrostomy tube placement for gastropexy. This has not previously been described in the literature. Unfortunately, our patient expired from unknown etiology. This lends an important question regarding whether a right hemicolectomy should have been done as he may have had anastomotic breakdown. As there are limited cases describing hepatic volvulus with subsequent bowel obstruction, it cannot be said what the best practice is.

## Conclusion

Hepatic volvulus is a seldom described pathology related to congenital absence of ligamentous development. Our case report depicts a 56-year-old male with a history of omphalocele and hepatic volvulus with bowel obstruction. He had a redundant falciform and congenital absence of triangular ligaments, resulting in a chronic, intermittent volvulus, and chronically dilated stomach and colon. We performed a hepatopexy, gastrostomy tube for gastropexy, and right hemicolectomy for risk reduction. Unfortunately, our patient passed away from unknown cause. Though we believe the hepatopexy was necessary, it does raise the question of the utility of performing a colectomy for risk reduction. Transverse colon volvuli are rare and have a high risk of mortality if they are not treated operatively. We believed it was in the patient’s best interest to perform the right hemicolectomy to prevent re-volvulus. The literature supports operative intervention on both pathologies though the standard of care is unknown. We aim to raise attention to our operative experience.
